# Predicting Time to Return to Cannabis Use After a Cessation Attempt: Impact of Cumulated Exposure to Nicotine-Containing Products

**DOI:** 10.1177/1179173X241259603

**Published:** 2024-06-05

**Authors:** Natalia Poliakova, Lydia A. Shrier, Sion Kim Harris, Richard E. Bélanger

**Affiliations:** 1 177453 Research Centre of CHU de Québec-Université Laval, Quebec City, QC, Canada; 2 1862 Boston Children’s Hospital and Harvard Medical School, Boston, MA, USA; 3 Faculty of Medicine, Université Laval, Quebec City, QC, Canada

**Keywords:** Cannabis, tobacco, nicotine, cotinine, co-use, cessation

## Abstract

**Objective:** Cannabis is frequently co-used with tobacco/nicotine products, especially among young adults. Little is known about the effects of this co-use on cannabis cessation outcomes. Within a sample of young adults using cannabis frequently (current use of ≥5 days/week in the past 3 months), this study aimed to (a) document sources of exposure to tobacco/nicotine products, whether used simultaneously with cannabis or on different occasions, (b) examine if the level of cumulated exposure to tobacco/nicotine (self-reported or from biochemical testing) could predict time to cannabis lapse during a cannabis abstinence period, and (c) explore the relationship between nicotine/tobacco exposure and time to cannabis lapse according to tobacco cigarette smoking status. **Method:** Urine cotinine measures and self-reported data on use of different tobacco/nicotine products, collected from 32 participants (aged 19 to 23), were analyzed to predict time to lapse during a 2-week period of attempted abstinence from cannabis, controlling for cannabis dependence and sex. **Results:** Half of participants (56.3%) used at least one tobacco/nicotine product. Higher urine cotinine, representing higher cumulated tobacco/nicotine exposure, was related to a higher risk of lapsing (Hazard Ratio [HR] = 1.64; 95%CI [1.04, 2.58]). The risk of lapsing was even higher ([HR] = 3.46; 95%CI [1.17, 10.25]) among heavily tobacco/nicotine exposed (>600 ng/mL, urine cotinine) participants than among unexposed (<50 ng/mL) or lightly/moderately exposed (50-600 ng/mL) participants. Among those smoking cigarettes (solely or in combination with other products), there was no relation between cotinine level and time to lapse, likely due to a reduced variability in abstinence probability and a high likelihood of lapse observed for higher cotinine levels, mainly achieved by cigarette use. **Conclusions:** With a rapidly changing landscape of tobacco/nicotine use, our results underscore the need to consider all sources of tobacco/nicotine exposure to fully understand the specific and cumulative contributions of tobacco/nicotine to cannabis cessation outcomes.

## Introduction

Worldwide, tobacco and cannabis rank among the most frequently used psychoactive substances, after alcohol.^1^ Notably, the United States (US) stands out with some of the highest levels of use of both substances.^2^ In 2020, 20.7% and 11.8% of Americans aged 12 and older reported past-month use of tobacco/nicotine products and cannabis, respectively.^3^ Globally, use of these substances raises particular concerns for young adults, as this group exhibits the highest cumulated incidence of use.^1^ In the US, people aged 18 to 25 comprise the largest percentage (25.1% and 23.1%, for tobacco/nicotine products and cannabis, respectively) of those reporting monthly use of the two substances.^3^

High prevalence of co-use of both substances has also been described and has become nearly normative among people using cannabis. For example, a cross-national study revealed that past-month use of at least one tobacco product was encountered among 69.7% of people using cannabis in Canada, US, and England.^4^ In another study, past-month tobacco use was reported by 68.6% of Americans aged 18 and older using cannabis.^5^ Notably, higher rates of combusted tobacco and polytobacco use are observed among those reporting cannabis use compared with those not using cannabis. In 2019, the prevalence of past-month co-use of cannabis with smoked tobacco or nicotine e-cigarette among U.S. adults was respectively 3.4% and 1.4% (34.5% and 15.4% among those using cannabis).^6^ Co-use is especially prevalent among young adults; 8 out of 10 18- to 24-year-olds (82.6%) using cannabis reported past-month tobacco use between 2014 and 2015.^7^ Co-use takes different forms where tobacco and cannabis are used either simultaneously (co-administration within the same product), sequentially (one substance used right after the other), or asynchronously (both substances used on different occasions).^8^ While sequential and asynchronous co-uses remain very common, co-administration of tobacco and cannabis by means of blunts (tobacco cigars filled with cannabis) or spliffs (joints filled with both tobacco and cannabis, also referred to as “mulling”) is increasing and represents a significant exposure to tobacco.^9^ In 2015-2016, the majority of young Americans (74.0%) ever exposed to cannabis reported lifetime use of blunts.^10^

Co-use of cannabis and tobacco is concerning because of health problems related to this use pattern. These health problems include a higher risk of heavier use of either substance or developing a use disorder, an increased likelihood of subsequent psychotic symptoms, and additive risks for toxicant exposure.^11,12^ Also, studies have highlighted poorer cannabis cessation outcomes among tobacco cigarette and cannabis co-users, in comparison to users of cannabis alone.^13-16^ Smoking tobacco cigarettes was the most important predictor of cannabis relapse within a sample reporting cannabis use.^15^ Similarly, Gray et al^14^ found that those smoking tobacco were half as likely as those who do not smoke to abstain from cannabis use during treatment, both in placebo and medicated groups. Moore and Budney^16^ observed poorer cannabis cessation outcomes for individuals smoking tobacco currently than for ex-smokers. In addition, regular cigarette smoking among adolescents was associated with less rapid decline in cannabis use when compared to non-regular cigarette smoking during a controlled trial targeting non-nicotine substance use disorders (SUD).^13^

Previous studies have concentrated primarily on the contribution of tobacco cigarette smoking to cannabis cessation outcomes. Even if tobacco cigarettes remain the most popular tobacco/nicotine product among those who co-use, recent data reveal great diversification in tobacco products co-used with cannabis^17^ due to rapidly changing public policies concerning both substances and an expanding number of available tobacco and cannabis products, as well as consumption devices. In the U.S. population, current cannabis use has been reported by 16% of individuals smoking exclusively tobacco cigarette, but also by notable proportions of those solely using electronic cigarettes (e-cigarettes; 15%), cigars (28%), hookah (29%), smokeless products (5%), as well as by people using multiple tobacco products (33%).^18^ In this context of diversification of co-use, whole understanding of cannabis cessation challenges and outcomes requires consideration of the contribution of overall tobacco/nicotine exposure, a contribution that remains currently unknown. Therefore, within a sample of young adults using cannabis frequently (current use of ≥5 days/week in the past 3 months), the objectives of this investigation were to (1) document sources of exposure to tobacco/nicotine products, whether used simultaneously with cannabis or on different occasions, (2) examine if the level of cumulative exposure to tobacco/nicotine (either self-reported or from biochemical testing) could predict time to cannabis lapse during a cannabis abstinence period, and (3) explore the relationship between nicotine/tobacco exposure and time to cannabis lapse according to tobacco cigarette smoking status. We hypothesized that, regardless of the source, higher cumulated nicotine exposure would be associated with poorer cannabis cessation outcomes. We did not formulate any specific hypotheses regarding the source of nicotine exposure. In case of significant associations, the results of this study will bring new insight into the contribution of tobacco/nicotine products to cannabis cessation outcomes and the necessity to consider all sources of tobacco/nicotine exposure in improving these outcomes.

## Method

### Sample

This investigation analyzed data from a community-recruited convenience sample of young adults using cannabis. We recruited participants by means of local twice-daily posted Craigslist Boston ads, targeting individuals using cannabis frequently (at least 5 days/week in the past 3 months), aged 18-25, who were able to travel to a hospital outpatient clinic in Boston for study visits, desiring and planning to quit, willing to abstain for two weeks, and not currently in cannabis use treatment. Exclusion/withdrawal criteria included a positive screen for hazardous alcohol use on the Alcohol Use Disorders Identification Test-Consumption,^19^ current dependence on substances other than cannabis or nicotine, and a recent change in usual routines. Of 105 young adults expressing interest, 55 were eligible, and 40 enrolled and completed the study. Six participants were withdrawn *a posteriori* due to baseline urinary 11-nor-9-carboxy-Δ^9^-tetrahydrocannabinol (THC-COOH) < 5 ng/mL and absence of use during the 2-week period before attempted abstinence. After exclusions, data from 34 participants were included in initial analyses. Following the assessment of the basic assumptions of the statistical analyses, two participants were removed from the initial sample (see also Section *Statistical analyses*). Consequently, the study sample consists of 32 participants, whose data were analyzed and reported in this study. A more detailed description of the sampling procedure and the characteristics of the initial sample is available elsewhere.^20^

### Procedures

This was a prospective cohort study conducted in 2015-2016 to identify momentary factors and individual characteristics related to risk of lapse during a period of attempted cannabis abstinence.^20^ At study entry, participants responded in person to a questionnaire on sociodemographic characteristics, substance use history, and cannabis use expectancies, motives, problems, and self-efficacy to abstain. We invited participants to maintain their usual substance use during the next two weeks followed by two weeks of attempted cannabis abstinence. Participants received no assistance or support for cannabis cessation. Measures related to cannabis use, such as craving, frequency of use or situational permissibility, were collected during these four weeks. After the phase of attempted abstinence, participants came to our lab to complete an exit questionnaire. Participants provided urine samples at study entry (baseline) and following the abstinence period (follow-up) for quantitative THC-COOH and cotinine analysis. Finally, participants received remuneration commensurate with completed study activities (≤ $250). The protocol was approved by the principal investigator’s (LAS) institutional review board. All participants provided written informed consent.

### Measures

#### Cannabis Use and Abstinence Outcomes

Participants reported on cannabis use history (age at initiation, age at first use weekly, and age at first use ≥3 times/week) and number of cannabis use occasions (range 1-99) during the week prior to the study enrollment. Cannabis use was documented during the 2-week attempted abstinence period using a Timeline Follow-back (TLFB) calendar, the most frequently used standardized instrument for measuring frequency of cannabis use.^21^ Validity studies have demonstrated that the TLFB can yield reliable information regarding cannabis use for up to a 12-month period preceding the interview,^22^ with an average agreement rate of 87.3% when compared to biological measures.^23^ Participants who reported on the TLFB no cannabis use during the period of attempted abstinence were considered abstinent. Time to lapse was defined as the number of days to the first cannabis use after the beginning of the abstinence period as reported on the TLFB.

Past-30-day cannabis dependence was assessed using the Severity of Dependence Scale (SDS) composed of 6 items on a scale ranging from *0* = *Never/Almost never* to *3* = *Always/Nearly always*, Cronbach α = .86.^24^ This tool compares favorably to more formal diagnostic assessments for cannabis dependence.^25^

#### Self-Reported Exposure to Nicotine-Containing Products

To document the baseline frequency of each smoking cigarettes, using other forms of tobacco (chewing tobacco, snuff, dip, cigars, cigarillos, little cigars), and using electronic nicotine vapor products (e-cigarette, e-cigar, e-hookah, vape pen) in the past 30 days, a closed-ended question with the response scale ranging from *0* = *0 day* to *6 = All 30 days* was employed. In addition, amount of exposure on use days was assessed for cigarette smoking and other tobacco products using the response scale ranging from *0 = Less than 1 cigarette per day/Less than one per day* to *5 = More than 20 cigarettes par day/More than 20 times per day.* Finally, past-month frequency of mulling (*0 = Always* to *4 = Never*) and the percentage of tobacco added (0% to 100%) were also assessed.

To estimate total exposure to tobacco/nicotine products from self-reported data, we created, for the purposes of this research, a new proxy variable, representing cumulative self-reported exposure with lower values corresponding to lower exposure and vice versa. This variable took into account both the reported frequency and quantity of a product used for each use. First, a variable representing the cumulated exposure to each of three sources (cigarette, other tobacco products, and mulling) was calculated by multiplying the frequency of one specific type of exposure (e.g., *1* = *1 or 2 days* per month smoking cigarettes) by amount of this exposure (e.g., *2 = 2 to 5 cigarettes per day*). Only exposure to electronic vapor products was represented by the question on the frequency of use. Second, all four variables were rescaled to range from 0-36 by dividing each score by an expected maximum and multiplying by 36. Finally, we summed all four variables to create a single variable of cumulated exposure.

#### Biochemical Testing of Exposure to Tobacco/Nicotine

Urine cotinine was used as a biomarker of cumulated exposure to tobacco and nicotine products. Urinary cotinine measurement is a valuable indicator of use of e-cigarettes and other tobacco and nicotine products.^26^ Urine baseline samples were aliquoted and frozen (−80°C) until batch testing. Cotinine concentrations were measured by an ELISA essay (Calbiotech Inc. Spring Valley, CA) using a competitive enzyme immunoassay technique. The assay demonstrates a sensitivity of .1 ng/mL, and day-to-day variability shows values of 8.9% at a concentration of 3.3 ng/mL (for non-smokers) and 5.0% at a concentration of 254.9 ng/mL (for smokers).^27^

### Statistical Analyses

Baseline urinary cotinine was analyzed both as a continuous variable and as a discrete variable categorizing tobacco exposure status based on the cotinine standard benchmarks: unexposed (<50 ng/mL) and exposed (≥50 ng/mL), with the latter group also subdivided into lightly to moderately exposed (50-600 ng/mL) and heavily exposed (>600 ng/mL).^26,28,29^

Descriptive analyses summarized demographic characteristics, cannabis and tobacco use for the entire sample and according to cotinine-based tobacco exposure status (currently unexposed vs exposed). The nonparametric Wilcoxon signed-rank test was used to compare measurements repeated over time. The Mann-Whitney *U* test and Fisher’s exact test for continuous and categorical variables, respectively, were used to compare participants according to their exposure status.

The association between cotinine and number of days to lapse were modeled using the multivariable Cox proportional hazard regression (PROC PHREG in SAS), adjusting for SDS, sex and highest school grade completed. Hazard ratios (HR) and the 95% confidence intervals (95% CI) were estimated. To assess the analysis assumptions, dfbeta values, Shoenfeld and Martingale residuals were graphically checked for influential observations, the proportional hazards assumption, and nonlinearity. Dfbeta values for two participants were high, exceeding .2*standard error of the parameter estimate. Therefore, to meet the assumptions of the Cox regression, data from these two participants were excluded from the analyses.

Data were analyzed using SAS 9.4 (SAS Institute Inc., Cary, NC, USA) with significance level set at a two-sided *P*-value <.05. Taking into consideration the exploratory nature of analyses, results with a *P*-value <.10 were also reported.

## Results

### Sample Characteristics and Baseline Cannabis Use

Demographic characteristics of the participants are reported in Table 1A. The median age was 22 years (interquartile range [IQR], 19.5-23.6), and nearly half of participants were male. Almost all were in school or working, with a majority completing at least some college, and approximately two-thirds of the participants were evenly distributed between White and Hispanic ethnicity.

**Table 1.** Sociodemographic Characteristics, Cannabis and Tobacco Products Use at Baseline and at the End of the Abstinence Phase for the Total Sample and for Participants Exposed and Unexposed to Tobacco Products.

Characteristic	Total (n = 32)	Recently Unexposed to Tobacco^a^ (n = 14)	Recently Exposed to Tobacco (n = 18)	P Value^b^
	Median (IQR)/%	Median (IQR)/%	Median (IQR)/%	
A. Sociodemographic and other characteristics				
Age, years	21.9 (19.5-23.6)	21.8 (19.5-22.3)	22.9 (19.7-24.7)	.31
Sex, male	47.0	46.0	52.5	.26
Race/Ethnicity				.33
Black	18.8	23.0	15.8	
White	31.2	46.2	21.1	
Hispanic	31.2	15.4	42.1	
Other or multi-race	18.8	15.4	21.1	
Occupation				.35
Studying	21.9	30.8	15.8	
Working	21.9	7.7	31.6	
Both	53.1	61.5	47.4	
None	3.1	0.0	5.2	
Highest school grade completed				.45
High school, GED or less	34.4	23.1	42.1	
At least some college	65.6	65.6	57.9	
B. Cannabis use				
Baseline past week use, number of times	11.0 (5.5-18.5)	5.0 (3.0-15.0)	12.0 (8.0-19.0)	.08
Severity of dependence scale				
Continuous (0-15)	4.0 (2.0-6.0)	6.0 (3.0-8.0)	3.0 (2.0-5.0)	.22
Dichotomous (≥4)	56.3	69.2	47.4	.29
Age at first use, years	15.0 (14.0-16.0)	16.0 (14.0-16.0)	15.0 (13.0-16.0)	.23
Age at weekly use, years	17.0 (16.0-18.0)	18.0 (16.0-18.0)	16.0 (16.0-18.0)	.27
Age at use ≥3 times/week, years	18.0 (16.5-18.5)	18.0 (17.0-19.0)	17.0 (16.0-18.0)	.12
Days to the lapse, number of days	2.0 (1.0-11.5)	11.0 (2.0-14.0)	1.0 (1.0-4.0)	**.01**
C. Tobacco/nicotine exposure at the baseline				
Cigarette use, at least 1-2 days in the last month	31.3	7.7	47.4	**.02**
Use of other forms of tobacco, at least 1-2 days in the last month	34.4	7.7	52.6	**.01**
Use of electronic vapor products, at least 1-2 days in the last month	21.9	23.1	21.1	1.00
Adding tobacco to smoked marijuana, at least 50% of the time	31.3	0.0	52.6	**.002**
Use of any tobacco/nicotine products, yes	56.3	23.1	79.0	**.003**

Notes. IQR, interquartile range. Value in bold indicate significant tests with a P value set at <.05.

^a^Unexposed to tobacco products group is defined as people having baseline urinary cotinine level lower than 50 ng/mL in comparison to the Exposed group with cotinine level of 50 ng/mL and higher.

^b^*t*-tests/the Mann-Whitney U test for continuous variables and Chi-square/Fishers exact test for categorical variables.

Cannabis use behaviour is reported in Table 1B. Half of participants met criteria for cannabis dependence using a cut-off of 4 on SDS total score. Frequency of cannabis use did not differ significantly (Wilcoxon *S* = 7.00, *P* = .898) from the week prior to the study enrollment (median [*Mdn*] = 11 times/week; IQR, 5.5-18.8) to the 2-week habitual use period (*Mdn* = 9.8 times/week; IQR, 8.0-13.0). Participants currently exposed and unexposed to nicotine did not differ on their sociodemographic characteristics or cannabis use-related variables other than days to lapse.

### Objective 1: Sources of Exposure to Nicotine-Containing Products

We documented use of four nicotine-containing products: (1) cigarettes, (2) other forms of tobacco (chewing tobacco, snuff, dip, cigars, cigarillos, or little cigars), (3) electronic vapor products (including e-cigarette, e-cigar, e-hookah, or vape pen), and (4) adding tobacco to smoked cannabis (see Table 1C). Approximately one-third of participants (31.3%–34.4%) reported exposure to each of the sources with the exception of electronic products use, which was reported by one-fifth of participants. Half of participants were exposed to at least one source of nicotine.

The created variable representing cumulative self-reported exposure was highly correlated with urine cotinine concentrations (see Table 2). When the sources of exposure were considered separately, cotinine was most associated with cigarette use and least associated with use of electronic vapor products. The sources of exposure were weakly and non-significantly correlated, except for mulling, which had a high association with use of other tobacco products and a moderate association with use of electronic products.

**Table 2.** Bivariate Correlations of Variables Related to Self-Reported Tobacco Use With Baseline Urinary Cotinine and Number of Days to Lapse.

	Cotinine (ng/mL)	Days to Lapse	1	2	3	4
Use of cigarette						
1.Total cigarette exposure (days*number of cigarettes), past 30 days	.54***	−.24	—			
Use of other tobacco products						
2.Total exposure to other tobacco products (days*number of times), past 30 days	.27	−.31	.13	—		
Use of electronic vapor product						
3.Number of days using an electronic vapor product, past 30 days	.08	.00	.29	.23	—	
Adding tobacco to smoked cannabis						
4.Total exposure to tobacco added (frequency*% added), past 30 days	.33	−.13	.17	.63***	.41*	—
Exposure to nicotine-containing products						
5.Cumulated exposure to cigarette, other products, electronic vapor products and tobacco added, past 30 days	.61***	−.28	.67***	.62***	.54**	.62***

*Note.* n = 32.

***P < .001; **P < .01; *P < .05.

### Objective 2: Cumulative (Biochemically Tested and Self-Reported) Exposure to Nicotine-Containing Products and Time to Cannabis Lapse

**Figure 1.**
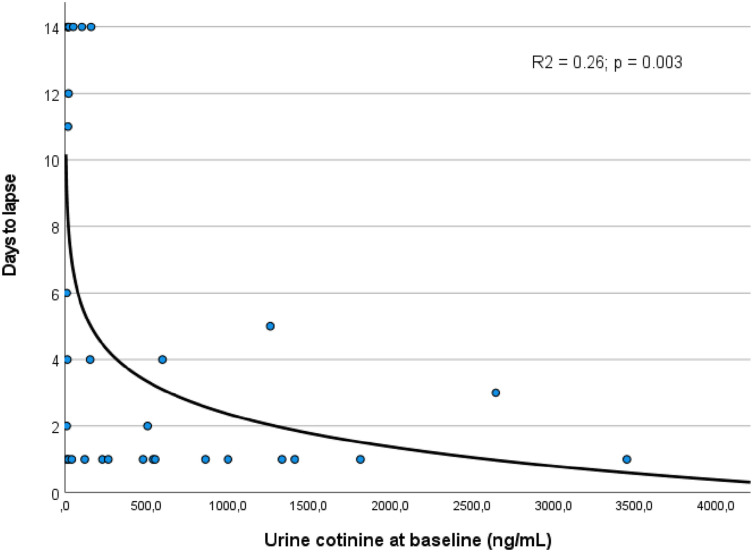
Relation between urine cotinine level at the baseline and number of days to lapse.

The lapse rate was high: 78.1% (25 of 32) of participants lapsed during the attempted abstinence period (*Mdn* = 2.0 days to lapse; IQR, 1.0-11.5). At the bivariate level, cotinine was logarithmically associated with period until lapse (*ß* = −1.43; *P* = .003; see Figure 1); approximately one-fourth of the variance observed in number of days to lapse was explained by urine cotinine level. After adjustment for SDS, sex and education of participants, we found a 64% increase in the risk of lapsing (HR, 1.64; 95% CI, 1.04-2.58; *P =* .04) during the attempted abstinence period with each increase of 1 mg per liter of cotinine (we changed the cotinine scale in this analysis to obtain a meaningful parameter estimate).

**Figure 2.**
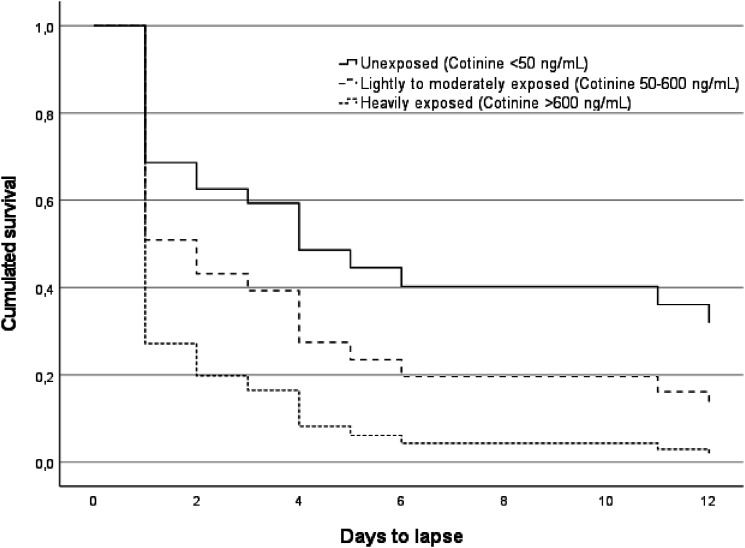
Predicted survival function for cannabis lapse according to levels of cumulated exposure to tobacco products, adjusted for severity of cannabis dependence and sex.

To examine probability of cannabis abstinence according to different levels of exposure, we compared three groups based on the cotinine standard benchmarks for unexposed (reference), lightly to moderately exposed (HR, 1.79; 95% CI, .66-54.87; *P =* .25) and heavily exposed participants (HR, 3.46; 95% CI, 1.17-10.25; *P =* .03). The estimated survival curves for days to cannabis lapse had similar between-group patterns (see Figure 2), but indicated that participants heavily exposed had a much steeper drop in survival probability at the beginning of the abstinence period than the unexposed or lightly/moderately exposed groups. We also examined if the number of days to lapse could be predicted by the variable representing cumulated self-reported exposure. This variable was not related to the lapse (HR, 1.03; 95% CI, 1.00-1.07; *P =* .07).

### Objective 3: Tobacco Exposure via Cigarettes vs Other Sources and Time to Cannabis Lapse

We included in the Cox regression model an interaction term of Cotinine*Source of exposure (cigarette alone or in combination with other products vs any other sources of exposure [electronic vapor products, other tobacco products, mulling]) to test if the relation between cotinine and days to lapse was different according to the source of exposure. Holding SDS, sex and education constant, cotinine level was not related to time to lapse among cigarette smokers (HR, 1.47; 95% CI, .69-3.13; *P =* .32) but significantly related among users of other sources (HR, 4.16; 95% CI, 1.12-15.45; *P =* .03), although the interaction term was not significant (Wald χ^2^ = 2.34; *P =* .13).

## Discussion

### Use of Tobacco Products by Cannabis Users

Consistent with national data on frequent tobacco use among individuals using cannabis,^5^ half of the young adults using cannabis participating in this study were exposed to at least one tobacco/nicotine product and a substantial proportion reported polytobacco use, with only one out of six tobacco/nicotine exposed participants using cigarettes exclusively (data not shown). Even if cigarette smoking is still very common among cannabis users,^5^ observed decreasing prevalence of cigarette use may reflect a transition to other forms of tobacco products. Concurrent with decreased rates of cigarette use, an increasing trend in blunt use was described among all cannabis users, especially among young and middle-aged adults.^5^ Also, the emergence of newer electronic nicotine delivery (END) products in the last decade, such as tanks or pod-mods, and their rapidly increasing use among adolescents and young adults are changing tobacco use patterns and have the potential to significantly affect co-use of tobacco and cannabis.^30^ For example, escalated END systems use was prospectively associated with cannabis use and with initiating marijuana vaping among US young adults.^31^ Likewise, an emerging population of young adults who predominantly vape cannabis and nicotine has been described.^32^ These results highlight the importance of considering all sources of tobacco/nicotine exposure, including emerging co-use patterns via vaping, when addressing cannabis use issues. Arguments for more exhaustive and proper monitoring of tobacco and cannabis co-use to better understand health effects of this behavior were also presented recently by Hindocha and McClure.^33^ Such information – currently very limited − is critical in the current context of gradual loss of popularity of cigarettes, diversification of tobacco/nicotine products, and methods of co-use.

### Cumulated/Specific Tobacco Exposure and Time to Cannabis Lapse

Previous studies highlighted the negative impacts of tobacco cigarette smoking on cannabis cessation outcomes.^13-16,34^ However, the impact of non-cigarette tobacco/nicotine products on cannabis use outcomes is still unknown. This study extends beyond the effect of cigarettes to reveal the contribution of cumulative nicotine exposure to a shorter time to cannabis lapse during a period of attempted abstinence. A rapid decline in time to lapse was observed at even very low levels of cotinine. In our sample, the lower levels of cotinine originated from sources of exposure other than cigarettes (see Figure A, Supplemental materials; mean levels of cotinine according to exposure sources). In agreement, lower levels of biomarkers of tobacco exposure were related to use of non-combustible tobacco products, such as e-cigarettes.^35,36^ Elsewhere, mulling also resulted in lower, but still substantial levels of cotinine, comparable to light or moderate cigarette smoking.^37^ Our results suggest that these alternative sources of exposure can considerably affect cannabis cessation outcomes and their contribution deserves further investigation.

We did not find a statistically significant relationship between cotinine and time to lapse among participants smoking tobacco cigarettes. However, this result does not mean that cigarette use does not matter for cannabis lapse. Quite the contrary, at higher levels of cotinine, achieved particularly by use of cigarettes (Figure A), there was virtually no variability in the probability of abstinence, with a very high likelihood of lapse. Given that cotinine levels in our study exhibited dependence on the source of tobacco/nicotine exposure, further studies employing large samples encompassing various levels of cotinine achieved from diverse tobacco and nicotine products will be essential for a deeper understanding of the relation between cotinine and time to lapse depending on particular tobacco/products.

### Biological vs Self-Reported Measures of Exposure

In addition to cotinine biomarkers, we examined the relation of the created measure of cumulated self-reported exposure with time to cannabis lapse. This relation approached but did not reach statistical significance. Studies on accuracy of self-reported measures of substance exposure are scarce and have presented mixed results that have varied according to the substance (tobacco vs cannabis) or the type of measure (user status vs amount of exposure).^38,39^ Furthermore, there is no consensus regarding the best approach to inquire the use of electronic vapor products, whose use patterns significantly differ from that of more traditional products, such as tobacco cigarettes.^40,41^ The accuracy of estimating exposure to electronic vapor products may depend on the manner of questioning its use. Finally, self-reported measures are subject to recall bias and social desirability, especially among certain sub-populations (e.g., pregnant persons) and in certain circumstances (e.g., context of treatment). Nevertheless, in our study, the created variable of cumulated self-reported exposure was highly correlated with cotinine. This suggests that self-reported measures could be a useful proxy of real exposure, especially when different sources of exposure are considered and when the risk of bias is reduced by using momentary assessment (reducing recall bias) and electronic data collection on a personal device (reducing socially desirable responding). Additional research is necessary to validate the algorithms used for creating cumulative exposure variables, ensuring that these variables effectively and accurately estimate total nicotine exposure from self-reported data.

Although the negative effects of tobacco smoking on cannabis cessation have garnered considerable empirical support, causal mechanisms are not clearly understood. We would like to mention a few potential and likely co-occurring explanations for our results. First, using the common liability theory,^8^ tobacco exposure and cannabis cessation outcomes may be related due to common genetic routes that trigger dependence on both substances.^42^ For example, cigarette dependence was reported more frequently by individuals using cannabis daily than by those who use it less frequently or abstain.^43^ However, it is important to highlight that in our study, cotinine predicted shorter time to lapse over and above the symptoms of cannabis dependence, and that nicotine dependence was low among the participants exposed to cigarettes (data not shown). Second, tobacco use may also enhance cannabis withdrawal symptoms and consequently increase risk of cannabis relapse. A recent meta-analysis described a higher prevalence of cannabis withdrawal syndrome among participants co-using tobacco.^44^ Different biological pathways, based mainly on nicotine’s effects on nicotinic acetylcholine receptors, have been suggested to explain increased cannabis withdrawal symptoms among those co-using tobacco and cannabis.^45^ Other studies suggested a complex interaction between the substances highlighting contribution of factors such as tobacco use history and level of dependence on both substances to the interplay between nicotine and cannabis.^46,47^ Third, as the level of cotinine was different according to the sources of exposure, it is also possible that the observed association could be explained by administration methods. In case of similar administration (e.g., smoked tobacco and cannabis), tobacco smoking may provide behavioral cueing, triggering cannabis use.^48^

The results of this study should be considered in light of some limitations. First, although we documented exposure to a wide range of tobacco products, we did not ask about use of blunts, or exposure to second-hand smoke which could affect the accuracy of cumulated self-reported exposure and its association with time to cannabis lapse. In addition, urinary cotinine reflects recent exposure to tobacco products and may be not representative of long-term exposure. However, our cotinine measures were highly correlated with self-reported data of cumulated past-30-day use. Cotinine level was also correlated more markedly with self-reported cigarette use in comparison with use of other tobacco/nicotine products. Therefore, this association could partially explain the observed relation between cotinine and time to cannabis lapse. We had significant results despite the small sample size. Nevertheless, this study’s results should be considered as exploratory and need to be replicated with larger samples. Finally, there may have been possible social desirability, selection, and recall bias. Future research with larger and more diverse samples is warranted.

## Conclusions

This study is among the first to describe the contribution of cumulated nicotine exposure to cannabis lapse during a period of attempted abstinence from cannabis. Any small cotinine increase, even at light levels of nicotine exposure, markedly reduced the probability of abstinence. The likelihood of abstinence was consistently low across higher levels of cotinine, achieved by use of tobacco cigarettes alone or in combination with other products. In the context of new products, devices and use methods, our results highlight the need to consider all sources of tobacco/nicotine exposure among individuals using cannabis and gather comprehensive data on cannabis-tobacco/nicotine co-use in order to understand specific and cumulated effect of different tobacco products, and to improve cannabis cessation outcomes.

## Supplemental Material

Supplemental Material - Predicting Time to Return to Cannabis Use After a Cessation Attempt: Impact of Cumulated Exposure to Nicotine-Containing Products Supplemental Material for Predicting Time to Return to Cannabis Use After a Cessation Attempt: Impact of Cumulated Exposure to Nicotine-Containing Products by Natalia Poliakova, Lydia A. Shrier, Sion Kim Harris and Richard E. Belanger in Tobacco Use Insights.
