# Supplementary material for: Predicting Time to Return to Cannabis Use After a Cessation Attempt: Impact of Cumulated Exposure to Nicotine-Containing Products
Source: Tob Use Insights. 2024 Jun 5;17:1179173X241259603. doi: 10.1177/1179173X241259603 (PMC11155328; doi:10.1177/1179173X241259603)
Supplement: Supplemental Material - Predicting Time to Return to Cannabis Use After a Cessation Attempt: Impact of Cumulated Exposure to Nicotine-Containing Products [file sj-pdf-1-tui-10.1177_1179173X241259603.pdf]

Supplementary material

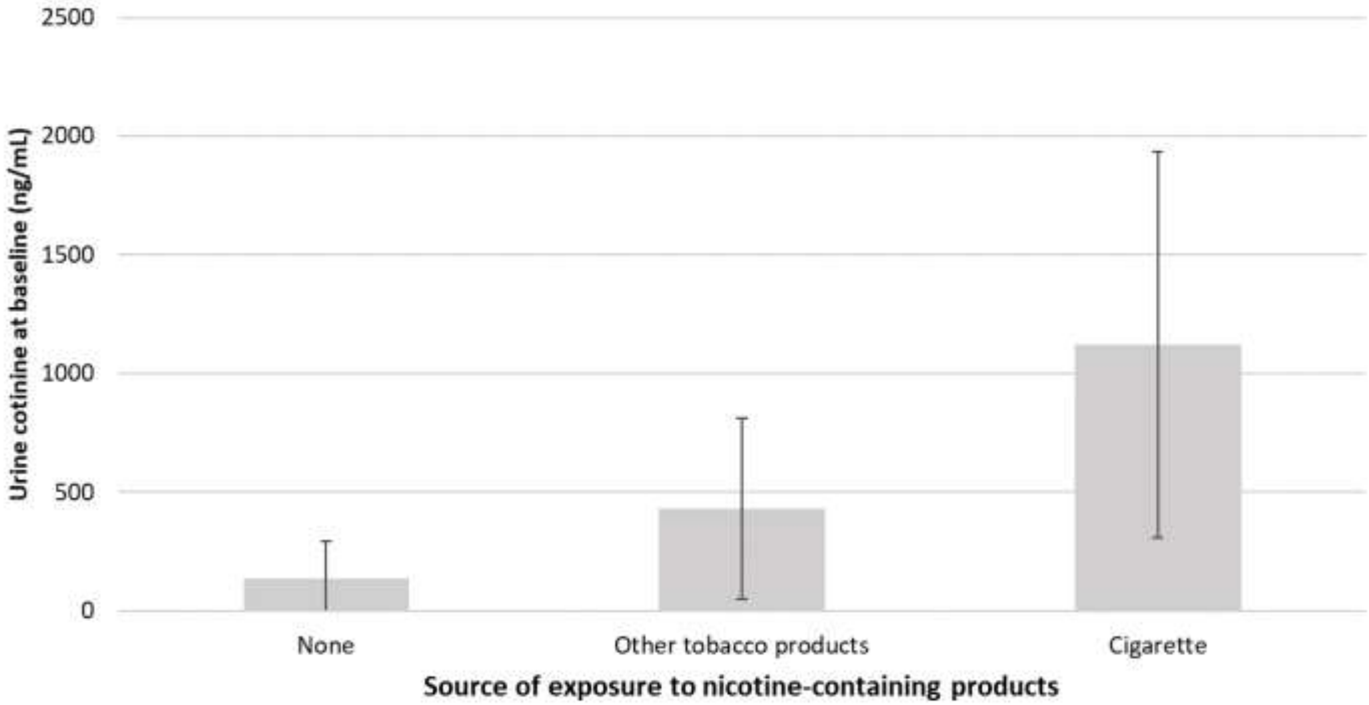

Figure A. Urinary cotinine levels at baseline according to the source of self-reported exposure to nicotine-containing products. Error bars represent standard errors.
